# Specific vulnerability of iPSC-derived motor neurons with TDP-43 gene mutation to oxidative stress

**DOI:** 10.1186/s13041-023-01050-w

**Published:** 2023-07-26

**Authors:** Asako Onda-Ohto, Minami Hasegawa-Ogawa, Hiromasa Matsuno, Tomotaka Shiraishi, Keiko Bono, Hiromi Hiraki, Yumi Kanegae, Yasuyuki Iguchi, Hirotaka James Okano

**Affiliations:** 1 Division of Regenerative Medicine, The Jikei University School of Medicine, 3-25-8 Nishi-Shimbashi, Minato-Ku, Tokyo, 105-8461 Japan; 2 Department of Neurology, The Jikei University School of Medicine, 3-25-8 Nishi-Shimbashi, Minato-Ku, Tokyo, 105-8461 Japan; 3 Core Research Facilities, Research Center for Medical Sciences, The Jikei University School of Medicine, 3-25-8 Nishi-Shimbashi, Minato-Ku, Tokyo, 105-8461 Japan

**Keywords:** Amyotrophic lateral sclerosis, iPSC, Motor neuron, Oxidative stress

## Abstract

Amyotrophic lateral sclerosis (ALS) is a disease that affects motor neurons and has a poor prognosis. We focused on TAR DNA-binding protein 43 kDa (TDP-43), which is a common component of neuronal inclusions in many ALS patients. To analyze the contribution of TDP-43 mutations to ALS in human cells, we first introduced TDP-43 mutations into healthy human iPSCs using CRISPR/Cas9 gene editing technology, induced the differentiation of these cells into motor and sensory neurons, and analyzed factors that are assumed to be altered in or associated with ALS (cell morphology, TDP-43 localization and aggregate formation, cell death, TDP-43 splicing function, etc.). We aimed to clarify the pathological alterations caused solely by TDP-43 mutation, i.e., the changes in human iPSC-derived neurons with TDP-43 mutation compared with those with the same genetic background except TDP-43 mutation. Oxidative stress induced by hydrogen peroxide administration caused the death of TDP-43 mutant-expressing motor neurons but not in sensory neurons, indicating the specific vulnerability of human iPSC-derived motor neurons with TDP-43 mutation to oxidative stress. In our model, we observed aggregate formation in a small fraction of TDP-43 mutant-expressing motor neurons, suggesting that aggregate formation seems to be related to ALS pathology but not the direct cause of cell death. This study provides basic knowledge for elucidating the pathogenesis of ALS and developing treatments for the disease.

**Supplementary Information:**

The online version contains supplementary material available at 10.1186/s13041-023-01050-w.

## Introduction

Amyotrophic lateral sclerosis (ALS) is a progressive disease characterized by motor neuron dysfunction, muscle weakness in the extremities, dysphagia, and respiratory impairment. ALS has a poor prognosis, with an average time from onset to death of approximately 3.5 years [1]. The cause of the disease has not yet been identified, and treatments for the disease are lacking. It is important to elucidate the pathogenesis of the disease to aid the development of treatments.

The majority of ALS cases are sporadic, and approximately 10% are familial cases [2, 3]. It is important to identify the causative gene of familial cases that show the same pathological changes as sporadic cases and to identify the protein that accumulates in the brain and spinal cord to elucidate the mechanism of degenerative diseases of unknown cause. Recent studies have identified TAR DNA-binding protein 43 kDa (TDP-43) as a component of neuronal inclusions in ALS, and many mutations in the *TARDBP* gene encoding TDP-43 have been reported. Interestingly, TDP-43-containing intraneuronal inclusions form in many ALS patients with and without *TARDBP* mutations, suggesting that determining the molecular mechanism of TDP-43 is key to elucidating the pathogenesis of ALS [4]. However, it is not clear how alterations in TDP-43 function are involved in ALS pathogenesis.

Seven TDP-43 mutants (G298S, A315T, M337V, Q343R, G348C, N352S, and A382T) were previously used to examine the relationship between TDP-43 half-life and age at onset of ALS, and the findings revealed that mutants with a longer half-life resulted in an earlier age at onset, i.e., there was a negative correlation between half-life and age at onset [5]. Among the seven mutants, A382T has a particularly long half-life and tends to result in a younger age of onset, suggesting that it is more toxic. Hasegawa et al. reported that TDP-43 upstream promoter activity is decreased by TDP-43 overexpression. The A382T mutant does not affect promoter activity, suggesting that the mutation may be responsible for the disruption of TDP-43’s self-regulatory function [6]. Based on these findings, we focused on the TDP-43 A382T mutant.

Transgenic and knockout mice have been used as animal models in the study of many neurodegenerative diseases. TDP-43 knockout mice are embryonic lethal [7]. Overexpression of either wild-type (WT) or mutant TDP-43 has been shown to be toxic to transgenic mice [8]. The difficulty in elucidating the pathogenesis of ALS is due to the lack of an appropriate disease model. Recent advances in genome editing technology have led to the development of Drosophila and mouse models with point mutations [9–11]. However, because these animal species differ from humans, it is unclear whether they accurately reflect human disease. Although patient-derived iPSCs are useful human disease models, it is difficult to attribute differences between control and mutant iPSCs because their genetic backgrounds are not identical. Moreover, because patient-derived iPSCs tend to show phenotypic variations, a large number of clones is needed for comparison with controls [12, 13].

This study elucidates the pathogenesis of ALS caused solely by TDP-43 mutation using human iPSC-derived motor and sensory neurons carrying the TDP-43 A382T mutation (introduced by CRISPR CAS9 gene editing technology) with the same background genes as healthy controls except for TDP-43.

The direct relationship between TDP-43-containing intraneuronal inclusions and motor neuron cell death in ALS patients is controversial [14–16], and pathological evaluation alone has limitations. In the present study, we examined the relationship between TDP-43-containing intraneuronal inclusions and motor neuron cell death**.**

## Results

### CRISPR CAS9 gene editing

**Fig. 1:**
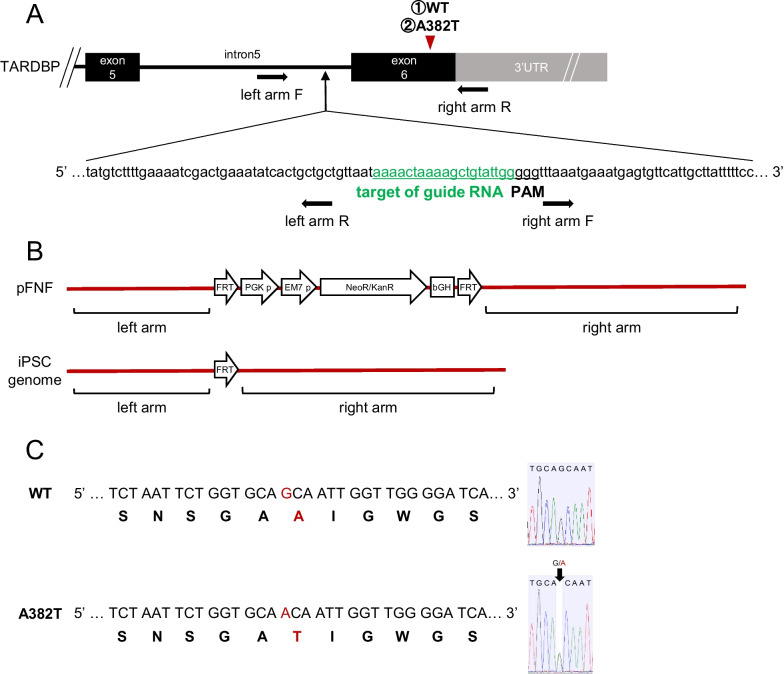
Genome editing by CRISPR/Cas9. **A** The A382T mutation was in exon 6 of the TARDBP gene, the guide RNA targeted intron 5 upstream of the mutation, and the left arm and right arm were designed. **B** FRT sequences were inserted into the iPSC genome after gene editing. **C** WT KI and A382T KI strains were established, and mutations were introduced into the genome of iPSCs

Using CRISPR CAS9 gene editing technology, we established a cell line with the A382T mutation. A single A382T allele was introduced into healthy human iPSC cells. Since FRT sequences are inserted during gene editing, we established a WT line expressing WT TDP-43 as a control for gene editing (Fig. 1). The established cell lines were confirmed to contain a heterologous mutation (c.1144G>A) in the genome sequence and have no off-target mutations or karyotype abnormalities (Additional file 1: Fig. S1A, B). In this study, the success rate of gene editing was 12.5–37.5% (1–3 out of 8 neomycin/puromycin-selected colonies).

### Differentiation of human iPSC-derived motor neurons and sensory neurons

**Fig. 2:**
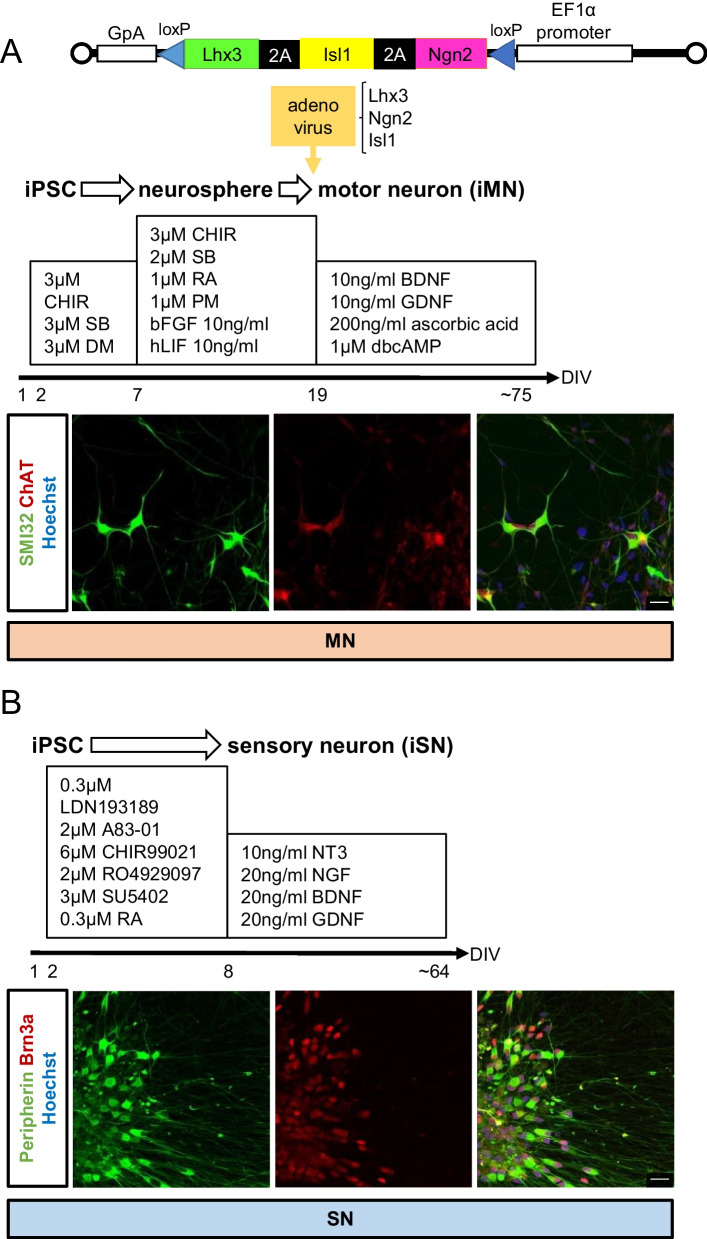
Differentiation of iPSCs into motor and sensory neurons. Schematic overview of the protocols for differentiation of iPSCs and immunostaining of iPSC-derived motor and sensory neurons. **A** Motor neuron; staining for the motor neuron markers SMI32 (green) and ChAT (red) is shown. **B** Sensory neuron; staining for the neuronal marker Peripherin (green) and the sensory neuron marker Brn3a (red) is shown. Scale bars, 20 μm in **A** and **B**

In this study, iPSCs were differentiated into motor and sensory neurons to verify whether the phenotypes caused by TDP-43 mutation are restricted to motor neurons. For motor neuron differentiation, iPSCs were maintained in Stem Fit medium containing CHIR99021, SB431542 and dorsomorphin for 6 days. We dissociated iPSCs into single cells and cultured these cells in neurosphere medium containing KBM, B27, CHIR99021, SB431542, retinoic acid, purmorphamine, bFGF and hLIF for 12 days to induce sphere formation (see “Methods”). The cells were induced to differentiate by using adenoviral vectors carrying three transcription factors, Lhx3, Ngn2, and Isl1. The efficiency of motor neuron differentiation was approximately 45%, as calculated by the ratio of ChAT-positive cells to cells with nuclear staining, indicating that this method was even more efficient compared to the conventional method using only small molecules [17, 18]. The sensory neuron differentiation protocol was modified from a previously reported method [19]. Specifically, we added reagents the day after iPSC passage instead of the day of passage, used Stem Fit medium instead of DMEM/F12 in the first half of the protocol, used KBM instead of DMEM/F12 in the second half of the protocol, and cultured the cells individually after 8 days of maintenance in the first half of the protocol followed by reseeding. The cells were cultured for up to approximately 60 days after differentiation. We confirmed the presence of cells positive for motor (SMI32, ChAT) and sensory (Brn3a) neuron markers (Fig. 2).

### Characterization of motor neurons and sensory neurons

**Fig. 3:**
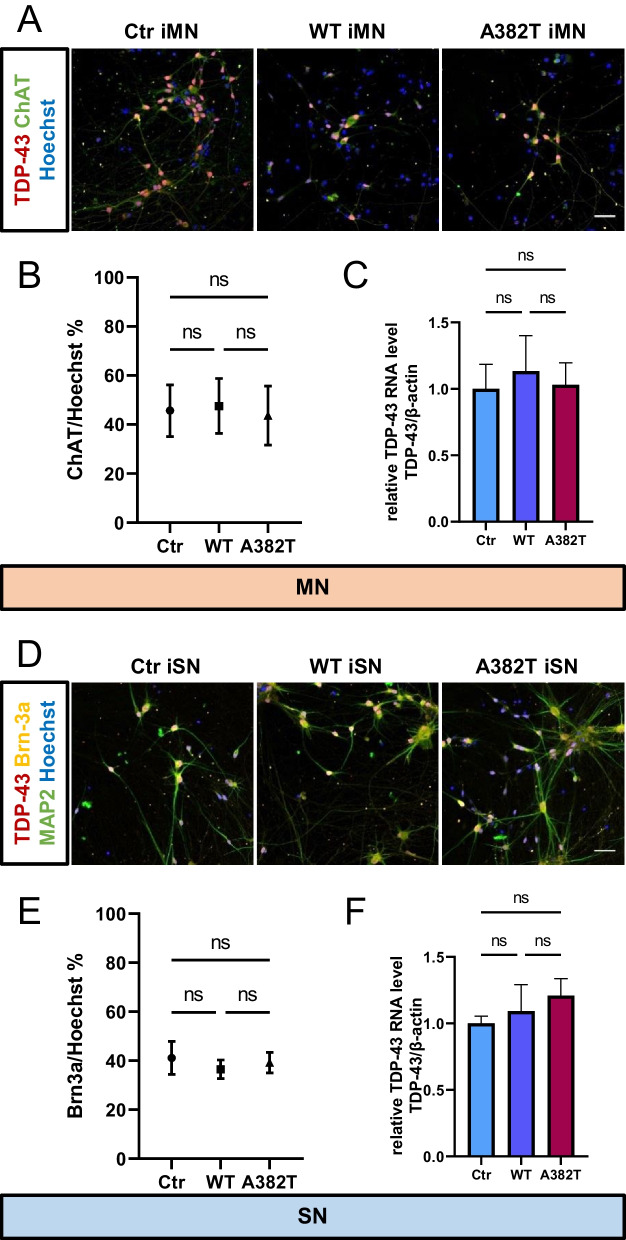
Evaluation of control and TDP-43 mutant-expressing neurons at approximately 60 days after seeding. **A**, **D** Three lines of unedited iPSCs and gene-edited iPSCs (WT and A382T lines) were differentiated into motor and sensory neurons. There were no differences in the morphology of motor neuron or sensory neuron bodies, axons, or dendrites among the three groups. TDP-43 was localized to the nucleus, and no aggregate formation was observed. **B**, **E** The efficiency of motor neuron differentiation was approximately 45%, as calculated by the ratio of ChAT-positive cells to cells with nuclear staining. The efficiency of sensory neuron differentiation was approximately 40%, as calculated by the ratio of Brn3a-positive cells to cells with nuclear staining. There were no significant differences in the percentage of cells positive for certain markers. **C**, **F** There were no significant differences in TDP-43 RNA levels in motor and sensory neurons among the three groups. Scale bars, 20 μm in **A**, **D**. **B**, **C**, **E**, **F** n = 3 in each of three groups. The data are presented as the mean ± SEM. n.s.: not significant. Statistical analyses were performed by one-way ANOVA

Three lines of unedited iPSCs (control) and gene-edited iPSCs (WT and A382T lines) were induced to differentiate into motor and sensory neurons. We evaluated the neurons at approximately 60 days after seeding. There were no differences in the morphology of motor and sensory neuron bodies, axons, or dendrites among the three groups. In ALS patients, TDP-43 is localized in the cytoplasm instead of the nucleus and forms aggregates in the cytoplasm. Immunostaining of cultured neurons showed that TDP-43 was localized to the nucleus, and no aggregates were observed (Fig. 3A, D). The motor neuron differentiation efficiency was approximately 45% in the control group, as calculated by the ratio of ChAT-positive cells to cells with nuclear staining. The sensory neuron differentiation efficiency was approximately 40% in the control group, as calculated by the ratio of Brn3a-positive cells to cells with nuclear staining. There were no significant differences in the percentage of cells positive for certain markers (Fig. 3B, E) or in TDP-43 RNA levels in motor and sensory neurons among the three groups (Fig. 3C, F). After approximately 60 days of incubation, no differences were observed in the disease groups.

### Evaluation of the effects of oxidative stress on motor neurons and sensory neurons

Evidence from both cell studies and clinical studies indicates the involvement of oxidative stress in neurodegenerative diseases, and we assessed whether oxidative stress induced by hydrogen peroxide causes neuronal injury using stress markers and cell death as indicators.

**Fig. 4:**
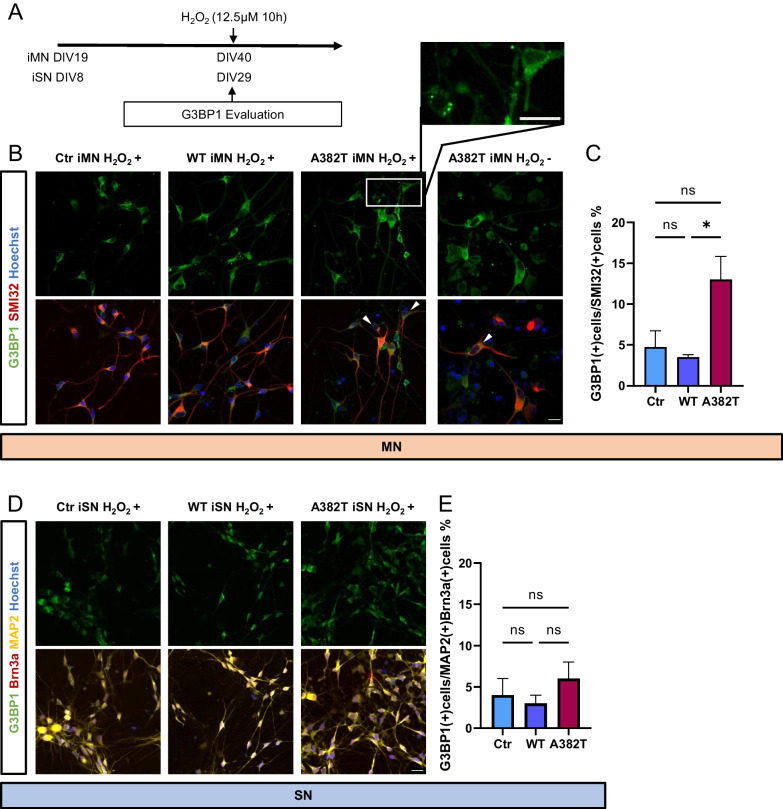
Evaluation of stress granule formation induced by oxidative stress. **A** Conditions of hydrogen peroxide administration and timing of G3BP1 expression evaluation. **B**, **D** Immunostaining of stressed neurons for the stress granule marker G3BP1. **C**, **E** Quantitative analysis of the percentage of stressed neurons. The percentage of stressed motor neurons was higher in the A382T group than in the control group. There were no significant differences in the percentage of stressed sensory neurons. Scale bars, 20 μm in **B**, **D**. **C**, **E** n = 5 in each of the three motor neuron groups. n = 3 in each of the three sensory neuron groups. The data are presented as the mean ± SEM. n.s., not significant, *P < 0.05. Statistical analyses were performed by one-way ANOVA

First, we evaluated the expression of G3BP1, a marker of stress granules. Stress granules are foci composed of RNA-binding proteins and RNA molecules. When cells are stressed, stress granules temporarily assemble to stop mRNA translation and induce the synthesis of cell-protective proteins to cope with cellular stress. Normally, they are evenly distributed in the cytoplasm, but under stress, they are observed as granules in the cytoplasm. Long-term exposure to low concentrations of hydrogen peroxide at 12.5 μM for 10 h tended to increase the proportion of G3BP1 granules in motor neurons expressing the A382T mutant (control vs. WT, P = 0.9297; control vs. A382T, P = 0.0782; WT vs. A382T, P = 0.0458), indicating hypersensitivity to stress. No significant difference in the ratio of G3BP1 granules in sensory neurons was found among the three groups (Fig. 4).

**Fig. 5:**
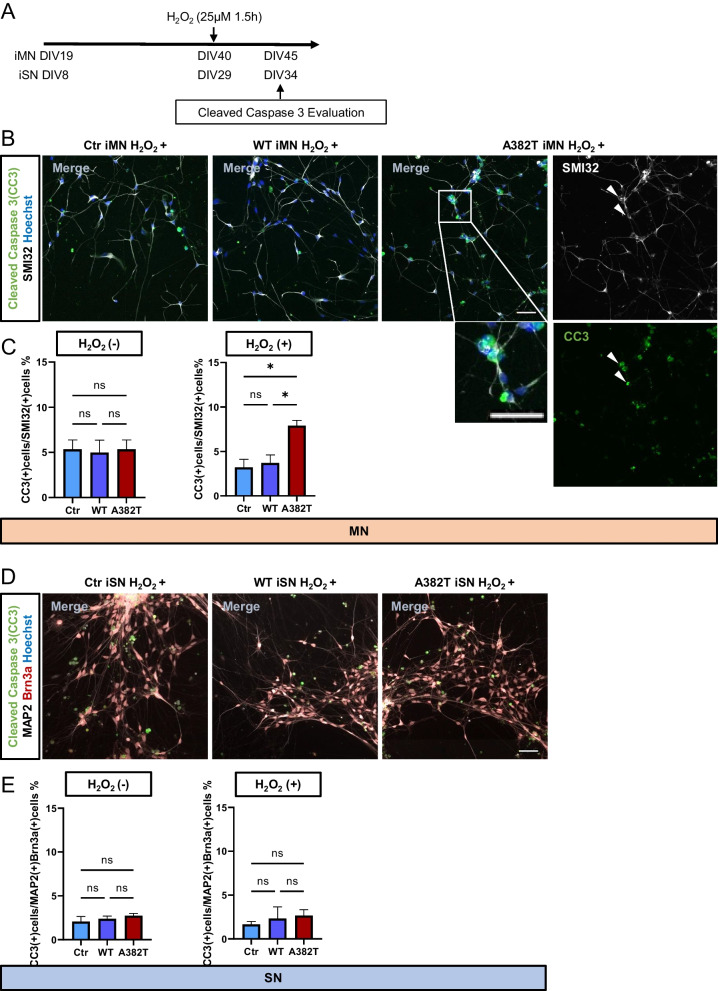
Evaluation of apoptosis induced by oxidative stress. **A** Conditions of hydrogen peroxide treatment and timing of CC3 expression evaluation. **B**, **D** Immunostaining of apoptotic neurons for the markers of apoptosis CC3. The SMI32/CC3-double positive cells in A382T iMN H_2_O_2_+ are indicated by arrowheads. **C**, **E** Quantitative analysis of the percentage of apoptotic neurons. The percentage of apoptotic motor neurons was higher in the A382T group than in the control group. There were no significant differences in the percentage of apoptotic sensory neurons. Scale bars, 20 μm in **B** and **D**. **C**, **E** n = 3 in each of the three groups. The data are presented as the mean ± SEM. n.s., not significant, *P < 0.05. Statistical analyses were performed by one-way ANOVA

Next, we evaluated the expression of cleaved caspase 3 (CC3), a marker of apoptosis. After exposure to 25 μM hydrogen peroxide for 1.5 h, the expression of CC3 was significantly increased in motor neurons expressing the A382T mutant compared to control and WT motor neurons (control vs. WT, P = 0.9037; control vs. A382T, P = 0.0156; WT vs. A382T, P = 0.0255). No significant difference in the expression of CC3 was observed in sensory neurons among the three groups (Fig. 5).

We next compared the length of neurites in the three groups to see whether hydrogen peroxide loading causes differences in neurite length since previous reports have indicated that the neurite retraction assay reflects early events of motor neuron degeneration in sporadic ALS iPSC-based model [12]. In same conditions as those used to evaluate CC3, we evaluated the total length of neurites/cell count of motor and sensory neurons at DIV45 and 34, respectively. The cells were evaluated separately as cleaved caspase3 negative or positive cells. There were no significant differences among the three groups in either (Additional file 2: Fig. S2A, B).

**Fig. 6:**
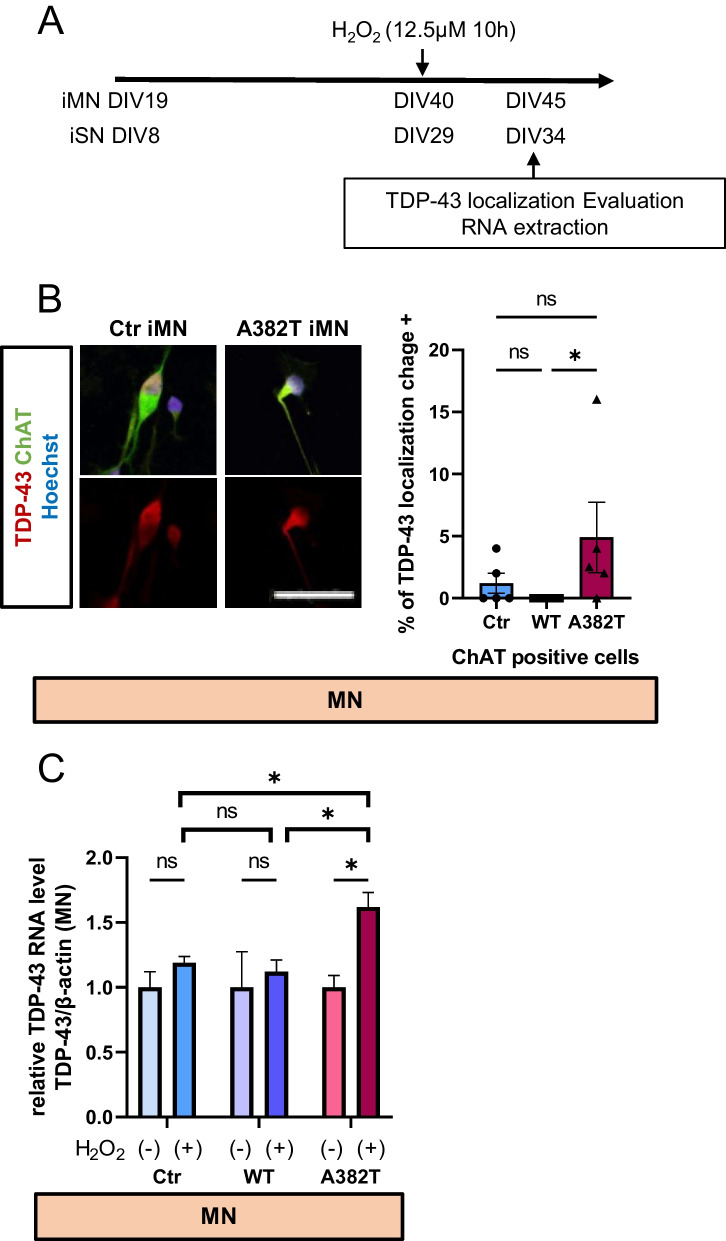
Evaluation of TDP-43 localization and TDP-43 RNA levels following oxidative stress. **A** Conditions of hydrogen peroxide treatment and timing of TDP-43 expression evaluation. **B** Immunostaining of TDP-43. Changes in TDP-43 localization were defined as positive when the cytoplasmic staining of TDP-43 was more intense than staining in the nucleus. Three experiments were performed, counting 50 cells per group per experiment. There was a trend toward an increase in the number of motor neurons in which TDP-43 localization was changed from the nucleus to the cytoplasm in the of A382T group. Scale bars, 20 μm. **C** RNA levels of TDP-43. TDP-43 RNA levels tended to increase in motor neurons in the A382T group when hydrogen peroxide was applied. **B** n = 5 in each of the three groups. The data are presented as the mean ± SEM. n.s., not significant, *P < 0.05. Statistical analyses were performed by one-way ANOVA. **C** n = 3 in each of the three groups. The data are presented as the mean ± SEM. n.s., not significant, *P < 0.05. Statistical analyses were performed by t test and one-way ANOVA

Furthermore, TDP-43 localization and TDP-43 RNA levels were assessed. After prolonged exposure to low concentrations of hydrogen peroxide (12.5 μM for 10 h), TDP-43 localization tended to shift from the nucleus to the cytoplasm in motor neurons expressing the A382T mutant compared to WT motor neurons (Fig. 6B). TDP-43 aggregate formation in the cytoplasm was observed rarely in motor neurons but only in those expressing the A382T mutant (Additional file 3: Fig. S3). Interestingly, in the absence of oxidative stress, there was no difference in TDP-43 RNA levels among the three groups, but when oxidative stress was induced, TDP-43 RNA levels tended to increase only in motor neurons expressing the A382T mutant (Fig. 6C). No changes in TDP-43 RNA levels were observed in sensory neurons (Additional file 4: Fig. S4).

Increasing the concentration of hydrogen peroxide or the duration of exposure resulted in cell death in all three groups (Additional file 5: Fig. S5).

The presence of the A382T mutant was confirmed in the genome in mutant iPSCs, but the mutation could not be confirmed in RNA in both A382T KI iPSC line-1 and A382T KI iPSC line-2 (Additional file 7: Fig. S7A). Similar results were also observed in hydrogen peroxide-treated neurons expressing the A382T mutant (Additional file 7: Fig. S7A). Considering the possibility that the mutant RNA was unstable and degraded, TDP-43 RNA and protein levels were evaluated, but there were no significant differences among the unedited control line, WT heterozygous line, WT homozygous line (established when the WT heterozygous line was created) and A382T line (Additional file 7: Fig. S7B, C).

### Evaluation of the splicing function of TDP-43

TDP-43 is an RNA-binding protein that is involved in the regulation of alternative splicing of target RNAs. PDP1 and BCL2L are known TDP-43 targets, and when TDP-43 function is impaired, the proportion of PDP1 splicing variants (PDP1-L/PDP1-S) increases, and the proportion of BCL2L variants (BCL2L-L/BCL2L-S) decreases. In this study, we evaluated the alternative splicing function of TDP-43 by collecting RNA the day after and 5 days after prolonged exposure to low concentrations of hydrogen peroxide. Evaluation of the splicing patterns of target RNAs revealed that TDP-43 function was not impaired by hydrogen peroxide, and there were no significant differences among the three groups (Additional file 6: Fig. S6).

## Discussion

Riluzole and edaravone have been approved to inhibit the progression of ALS, and a high-dose mecobalamine regimen is pending approval [20]. Recent studies have shown that both ropinirole and bosutinib have some therapeutic effects in suppressing the progression of ALS [21, 22]. For further development of drug discovery in the future, appropriate neuronal models are needed. Because ALS takes many years to develop, it is difficult to accurately recapitulate the clinical course of the disease in vitro. Therefore, the generation of neuronal models in which stress induces cell death is desirable.

Although studies have analyzed ALS patient-derived iPSCs [12, 13, 23–25] it has not been verified whether the observed alterations in these cells are caused by genetic mutations due to the diversity of the patients’ genetic backgrounds. There have been studies in which mutations in ALS patient-derived iPSCs were corrected by gene editing [26] and studies in which SOD1 mutations, which have been found in ALS, were introduced into healthy human-derived iPSCs [27]. This study is the first attempt to introduce TDP-43 mutations into healthy human iPSCs, and comparison with control cells with the same genetic background gene allowed us to elucidate the molecular pathogenesis of ALS related to the TDP-43 mutation. In this study, we established WT and A382T mutant-expressing cell lines by introducing normal TDP-43 and mutant TDP-43 (A382T) into healthy human iPSCs using CRISPR CAS9 gene editing technology. Control cells, WT cells and A382T mutant cells were differentiated into neurons, and morphological and functional evaluations were conducted.

While ALS is generally considered to specifically affect motor neurons, 32% of ALS patients have sensory impairment, and there are reports of axonal and demyelinating degeneration on peroneal nerve biopsy [28] and abnormal TDP-43 expression in cortical, subcortical, and thalamic areas, including sensory areas, on autopsy [29], indicating that sensory neurons in addition to motor neurons may degenerate [30, 31]. In addition, sensory neurons from mice with TDP-43 (A315T) or SOD1 (G93A) mutations show slower neurite outgrowth, fewer neurite branches, and greater sensitivity to vincristine, a microtubule inhibitor that causes axonal degeneration, than control neurons [32]. In this study, motor neurons and sensory neurons were derived from iPSCs to verify whether the changes caused by TDP-43 mutation are restricted to motor neurons.

There were no differences in the expression of neuronal markers or the morphology of motor and sensory neuron bodies, axons, or dendrites among the three groups after 2 months of culture. TDP-43 localization changes and aggregate formation, which are seen in affected tissues from ALS patients, were not observed.

Hydrogen peroxide has been used to induce oxidative stress in various studies; specifically, hydrogen peroxide resulted in a reduced neurite length and electrophysiological deficits in iPSC-derived motor neurons [33]. Hydrogen peroxide-treated motor neuron-like (NSC34) cells exhibited decreased survival and increased ROS levels [34]. Hydrogen peroxide administration resulted in a dose-dependent decrease in the survival of mutant SOD1-expressing NSC34 cells [35]. In this study, oxidative stress induced by hydrogen peroxide resulted in the formation of stress granules and eventual apoptosis in TDP-43 mutant-expressing motor neurons but not in sensory neurons. Motor neuron-specific vulnerability was demonstrated using iPSC-derived motor and sensory neurons expressing mutant TDP-43. Although the ratio of apoptosis in A382T was consistently and significantly higher than that of Control or WT with the oxidative stress load, the appropriate concentration and duration of exposure to hydrogen peroxide in this study were difficult to set up. Since not only the mutant-expressing but also control motor neurons were extremely sensitive to hydrogen peroxide, the most important step of this analysis was to always compare among the genotype-groups in each experiment. We are currently examining the possibility of using a different kind of stress load, which may result in more obvious differences in CC3 assay.

There was a trend toward a shift from nuclear to cytoplasmic localization of TDP-43 in TDP-43 mutant-expressing motor neurons and TDP-43 aggregate formation in a small fraction of TDP-43 mutant-expressing motor neurons. ALS patients show changes in TDP-43 localization and aggregate formation in motor neurons and motor neuron loss in the final stages of the disease, but whether aggregate formation is directly related to cell death is controversial. In our model, we observed aggregate formation in a small fraction of TDP-43 mutant-expressing motor neurons, suggesting that aggregate formation seems to be related to ALS pathology but is not the direct cause of cell death. In addition, we hypothesized that the increase in TDP-43 RNA levels in TDP-43 mutant-expressing motor neurons under oxidative stress may have been due to feedback resulting from a decrease in nuclear TDP-43 protein levels resulting in the change in TDP-43 localization from the nucleus to the cytoplasm.

Previous reports have suggested that motor neurons are vulnerable in ALS because they are larger than other cells; require large amounts of energy to maintain homeostatic ionic gradients and generate action potentials; are easily overloaded [36]; and have a lower buffering capacity for glutamate-triggered calcium ion influx [37], decreased expression of calcium-binding proteins such as CalbindinD28K and parvalbumin [38, 39], and decreased expression of GABAA receptor α1, which is involved in inhibitory synaptic transmission [40, 41]. TDP-43 mislocalization from the nucleus to the cytoplasm is more common in neurons than in other cells in ALS [42], and the half-life of mutant TDP-43 is longer than that of WT TDP-43 [5], suggesting that alterations in TDP-43 function may enhance motor neuron vulnerability in ALS.

We also evaluated the splicing patterns of PDP1 and BCL2L, alternative splicing targets of TDP-43, before and after stress and found no evidence of a stress-induced loss of TDP-43 function. The expression of ATG4B, GPSM2 [43], UNC13A [44] and STMN2 [45], which are targets of the TDP-43 cryptic exon indicating a loss of TDP-43 function, was also evaluated, but no cryptic exons were found (data not shown). In vivo studies of sporadic and familial ALS have shown the presence of cryptic exons and altered patterns of alternative splicing, indicating reduced TDP-43 function [9, 43, 46]. This is the first attempt to evaluate TDP-43 function in vitro in sporadic and familial ALS. We found no findings suggestive of TDP-43 hypofunction.

In clinical practice, it is difficult to assess the early changes that occur in ALS patients autopsy is an evaluation of the late stages of the disease. Although the present study demonstrated motor neuron-specific vulnerability to stress, cell death occurs in only a small percentage of neurons, and there was no loss of TDP-43 function. This may be an early response to ALS. In this study, hydrogen peroxide was used to induce very mild oxidative stress, which does not normally cause a response but can cause vulnerability in the presence of mutations or other predisposing factors. Aging, which is considered a major risk factor for neurodegenerative diseases [47, 48], may reflect prolonged exposure to minor stresses, and we considered our model to mimic what is observed in clinical practice. In the present study, it was difficult to evaluate under the condition of hydrogen peroxide for longer period and at higher concentrations, which induced the death of all cells regardless of whether they were healthy or diseased. In the future, it may be possible to develop a more accurate ALS-like model by using other stressors, such as arsenic and glutamate, or using organoids [49, 50] which can be cultured for a long period of time. In addition, RNAseq analysis on the neurons generated in this study may bring us closer to elucidating the pathophysiology of ALS.

The discovery of therapeutic drugs for ALS requires a neuronal model that closely resembles the motor neurons in ALS patients. In the present study, we developed a model in which prolonged exposure to oxidative stress specifically induced the death of motor neurons derived from human iPSCs expressing mutant TDP-43 and observed the vulnerability of motor neurons to stress, which is clinically relevant. The results of this study provide basic knowledge for elucidating the pathophysiology of ALS and developing novel treatments for the disease by drug screening using these neurons.

## Methods

### iPSC culture

iPSCs were maintained in culture plates coated with iMatrix-511 (Nippi) in Stem Fit medium (Ajinomoto) under feeder-free conditions and passaged every 4–7 days by 0.5× TrypLE Select Solution (CTS TrypLE Select Enzyme; Gibco, 0.5 M EDTA pH 8.0; Life Technologies, D-PBS(-); FUJIFILM WAKO) [51, 52].

### CRISPR/CAS9-mediated genome editing

The flow chart of the CRISPR Cas9-mediated gene editing protocol is shown in Fig. 1. The CRISPR direct web tool [53] was used to design a guide RNA; the left arm was upstream of the guide RNA, and the right arm was downstream of the guide RNA. The primers used for the left arm, right arm, and guide RNA are shown below. The plasmids used in this study, pFNF, px330-mcherry, and pCAGGS-flpE-puro, were purchased from Addgene. pFNF was inserted into the left arm and right arm sequences, and px330-mcherry was inserted into the guide RNA sequence. The restriction enzyme BbsI was used to insert the guide RNA. However, Bbs1 was also present in mCherry, and mCherry was cleaved, resulting in inefficient reconstruction. Therefore, we used the fluorescent dye Venus instead of mCherry. The plasmids were transfected using FuGENE HD transfection reagent (Promega).

Introduction of mutant TDP-43 (A382T) into a healthy control iPSC line (201B7) was carried out by CRISPR‒Cas9 genome editing. iPSCs were passaged, and px330 and pFNF were transfected into the cells. The cells were sorted with neomycin (f.c. 50 µg/ml) because the cells acquired neomycin resistance after successful transfection. At the next passage, pCAGGS-flpE-puro was transfected into the cells, and neomycin resistance was removed using the Flp-FRT system. Puromycin (f.c. 2 µg/ml) was used to sort the cells because successful expression of the Flp-FRT system caused the cells to transiently acquire puromycin resistance. Surviving colonies were selected, and cell lines with mutations in one allele were established by target region PCR. We established two cell lines, the WT cell line, in which WT TDP-43 was knocked in, and the A382T cell line, in which the mutant sequence was knocked in. Genomic DNA was extracted from the established cell lines using the QIAamp DNA Mini Kit (QIAGEN), and the insertion of the correct sequence was confirmed as follows.

- Left arm-F 5′–3′ GCCACCACGCCTGGCTGATTTTTTTG
- Left arm-R 5′–3′ GCAGCAGTGATATTTCAGTCGATTTTC
- Right arm-F 5′–3′ TTTAAATGAAATGAGTGTTCATTGC
- Right arm-R 5′–3′ CAGTTCACTTTCAACCACTC
- Guide RNA
- 5′–3′ CACCGaaaactaaaagctgtattgg
- 5′–3′ AAACccaatacagcttttagttttC

### Off-target analysis

By using the COSMID web tool [54], we selected five candidate sequences for analysis of possible off-targets. Using genomic DNA extracted from iPSCs, we amplified the region around the possible off-target site by PCR and confirmed the sequence of the PCR product. No off-target mutations were found in either the WT or A382T line. The primers used are shown below.

- Off target 1-F 5′–3′ GGGAAACAAGAAACTGAGGTGTGG
- Off target 1-R 5′–3′ CAGCTTGTTTTGCCTTCAGGGCTT
- Off target 2-F 5′–3′ GCAAATCTGTCCTTTGTGGGGG
- Off target 2-R 5′–3′ GCCAAGCAGCAAGACAAAAAGGG
- Off target 3-F 5′–3′ GAGACTTTCTCCCTGTCAGACAGC
- Off target 3-R 5′–3′ TGTCACCCTCTTTTACCATACGCC
- Off target 4-F 5′–3′ GGATTGCCTTTTTGATTTGGTCATCAGCTAG
- Off target 4-R 5′–3′ CAGCCAACATCACACTTAGTGGAG
- Off target 5-F 5′–3′ AGCAGCCTTAGGTAAGAGGTAGTG
- Off target 5-R 5′–3′ CCTCAAATTCCTGGGCTCAAGTG

In addition, karyotyping analysis was performed by Nihon Gene Research Laboratories, Inc., Japan.

### Differentiation of iPSCs into motor neurons

iPSCs were maintained in Stem Fit medium containing 3 μM CHIR99021 (Cayman Chemical), 3 μM SB431542 (Sigma‒Aldrich), and 3 μM dorsomorphin (Santa Cruz) for 6 days from the day after passage. The cells were dissociated into single cells with TrypLE Select (Gibco) and passaged in a flask in neurosphere medium consisting of KBM neural stem cell medium (KOHJIN Bio) containing 2% B27 supplement (Gibco), 3 µM CHIR99021, 2 µM SB431542, 1 µM retinoic acid (Wako), 1 µM purmorphamine (Cayman Chemical), 10 ng/ml basic fibroblast growth factor (Wako), 10 ng/ml human leukemia inhibitory factor (Millipore), and 10 µM Y-27632 (Wako) to induce sphere formation. The medium was changed on day 3. Neurospheres were passaged on day 6, and the medium was replaced on day 9 with neurosphere medium containing all of the components described above except Y-27632. On day 12, cells in neurospheres were dissociated into single cells with TrypLE Select, replated on plates coated with poly-l-ornithine (Sigma-Aldrich) and laminin (Invitrogen) at a density of approximately 0.5 to 1 × 10^5^ cells/cm^2^ and incubated with adenovirus vector (AdV) carrying three transcription factors, Lhx3, Ngn2 and Isl1, at a multiplicity of infection (MOI) of approximately 15. The Ngn2-, Isl1- and Lhx3-expressing adenovirus vector was constructed using the cosmid cassette pAxEFwit2, which has a cloning site SwaI flanked by the EF1 alpha promoter and rabbit beta-globin polyA signal in the E1-deleted region of the adenovirus genome. Each cDNA was ligated with 2A sequences derived from porcine Teschovirus for expression in the same vector. The AdV was prepared as previously described [55]. The AdV was titrated using the methods described by Pei et al. [56]. Briefly, the copy number of a viral gene that was successfully transduced into infected target cells was measured using qPCR (relative virus titer: rVT). After seeding, motor neurons were cultured with neuronal medium consisting of KBM neural stem cell medium containing 2% B27, 10 ng/ml brain-derived neurotrophic factor (BDNF) (R&D Systems), 10 ng/ml glial cell line-derived neurotrophic factor (GDNF) (R&D Systems), 200 ng/ml ascorbic acid (FUJIFILM WAKO) and 1 μM dibutyryl cyclic adenosine monophosphate (Sigma‒Aldrich). The medium was changed every 2–3 days (in preparation).

### Differentiation of iPSCs into sensory neurons

For sensory neuron differentiation, a previously reported method was used with modifications [19]. iPSCs were maintained in Stem Fit medium containing 0.3 μM LDN193189 (Stemgent), 2 μM A83-01 (Wako), 6 μM CHIR99021, 2 μM RO4929097 (Cellagen Technology), 3 μM SU5402 (TOCRIS), and 0.3 μM retinoic acid for 8 days from the day after passage. The cells were dissociated into single cells with 0.5× TrypLE Select Solution and replated on plates coated with poly-l-ornithine and laminin at a density of approximately 0.5 × 10^4^ cells/cm^2^ to induce differentiation into sensory neurons. After seeding, the sensory neurons were cultured with neuron medium consisting of KBM neural stem cell medium containing 2% B27, 10 ng/ml neurotrophin-3 (R&D Systems), 20 ng/ml NGF (R&D Systems), 20 ng/ml BDNF, and 20 ng/ml GDNF. The medium was changed every 2–3 days.

### Immunocytochemistry

Cells were fixed in phosphate-buffered saline (PBS) containing 4% paraformaldehyde (PFA) for 5 min at room temperature. After permeabilization and blocking with 0.3% Triton X-100 and 5% bovine serum albumin (BSA) for 60 min, the cells were incubated with primary antibodies at 4 °C overnight. Primary antibodies against the following were used for analyses: ChAT (goat, 1:50, Millipore), SMI32 (mouse, 1:500, Bio Legend), Brn3a (mouse, 1:50, Santa Cruz), Peripherin (mouse, 1:500, Santa Cruz), MAP2 (chicken, 1:10,000, Abcam), MAP2 (mouse, 1:100, Sigma‒Aldrich), Cleaved Caspase 3 (rabbit, 1:400, Cell Signaling Technology), G3BP1 (rabbit, 1:1000, Invitrogen), TDP-43(N) (rabbit, 1:100, Proteintech). The following day, the cells were washed with PBS and incubated with secondary antibodies for 60 min at room temperature. The secondary antibodies used were goat or donkey antibodies conjugated to Alexa 488, 546, 555, 568, 594, 633, or 647 (1:200; Invitrogen). Nuclear staining was performed with Hoechst 33342 (1:1000; Invitrogen) with secondary antibodies. Images were obtained using a confocal laser microscope (LSM880, Carl Zeiss).

### Oxidative stress induction

Hydrogen peroxide (H_2_O_2_, 30% w/v, Wako) was administered on day 21 of neuron culture. The expression of CC3, a marker of apoptosis, was assessed after 5 days of exposure to 25 μM H_2_O_2_ for 1.5 h. The expression of G3BP1, a marker of stress granules, was assessed after exposure to 12.5 μM H_2_O_2_ for 10 h. TDP-43 localization was evaluated after 5 days of exposure to 12.5 μM H_2_O_2_ for 10 h. RNA was extracted using TRIzol (Life Technologies) and an RNeasy Plus Mini Kit (QIAGEN) on the day after and 5 days after exposure to 12.5 μM H_2_O_2_ for 10 h. A total of 3 independent experiments were carried out.

### Reverse transcription polymerase chain reaction (RT-PCR) and evaluation of alternative splicing targets of TDP-43

cDNA was synthesized from total RNA extracted from cultured cells by ReverTra Ace (TOYOBO). Numerous primers were designed against alternative splicing targets of TDP-43 [57, 58]. The primers are listed below:

- PDP1-F 5′–3′ GCGTGGAAAGAGCGCCGAGC
- PDP1-R 5′–3′ TGCAGTGCCATAGATCCTGC
- BCL2L-F 5′–3′ TCTGAGTGTGACCGAGAAGG
- BCL2L-R 5′–3′ TCTTGGGCGATCCATATCTC

As controls for the evaluation of splicing function, control siRNA and siRNA targeting TDP-43 were transfected into HEK293T cells. Lipofectamine RNAiMAX (Life Technologies) was used for transfection of siRNAs. The interference efficiency was evaluated by PCR amplification of cDNAs in the target region. The sequences of the siRNAs were previously reported [6].

### Real-time RT-PCR

Real-time RT-PCR was performed using TaqMan Fast Advanced Master Mix (Thermo Fisher Scientific) with a Quant Studio 5 Real-time PCR system (Thermo Fisher Scientific). TDP-43 mRNA levels in the samples were normalized using beta-actin (TaqMan Gene Expression Assay: Assay ID: Hs01060665_g1). Probes and primers for TDP-43 were designed. The sequences were as follows:

- TDP-43 TaqMan probe design 5′–3′ AAAAAGGAAGAGCTAAAGGA
- TDP-43-F 5′–3′ TTTGTTCAGTGTGGAGTATATTCAGCA
- TDP-43-R 5′–3′ AACCACTCAATATTTCAACCTTTCATG

### Western blotting

Protein extraction from cultured iPSCs and neurons was performed using a mixture of Complete Mini (Sigma-Aldrich), phosphatase inhibitor (Nacalai Tesque) and Tissue Extraction Reagent I (Invitrogen) consisting of 50 mM Tris (pH 7.4), 250 mM NaCl, 5 mM EDTA, 2 mM Na_3_VO_4_, 1 mM NaF, 20 mM Na_4_P_2_O_7_, and 0.02% NaN_3_, which was added to each well on ice. The samples were collected in tubes, vortexed for 2 s, and then sonicated for 20 s. Centrifugation was performed at 15,000 rpm for 5 min at 4 °C, and the lysates were stored at − 80 °C. The samples were diluted with equal volumes of Laemmli sample buffer (Bio-Rad) containing 62.5 mM Tris–HCl (pH 6.8), 25% glycerol, 2% SDS, 0.01% bromophenol blue, and 5% 2-mercaptoethanol and then incubated for 3 min at 95 °C. Protein samples were separated using SDS-PAGE and transferred to polyvinylidene difluoride membranes (Millipore). The membranes were blocked with 1% nonfat skim milk for 30 min at room temperature (RT) and incubated in primary antibody for 16 h at 4 °C. Thereafter, the membranes were incubated with secondary antibodies for 2 h at RT. Signals were detected using Chemiluminescence HRP Substrate (Takara Bio) with a Lumino Graph I system (ATTO). Semiquantitative analysis of the signals was performed using ImageJ software (National Institute of Health).

### Statistical analyses

Statistical analyses were performed using a commercially available software package (GraphPad Prism 9). The data are presented as the mean ± SEM or SD as indicated. Statistical significance was assessed using Student’s t test or one-way ANOVA (*P < 0.05, **P < 0.01, ***P < 0.001). The number of independent experiments (n) is indicated in each figure legend.

## Supplementary Information

**Additional file 1: Figure S1.** (A) Off-target analysis of WT KI and A382T KI iPSCs. (B) Karyotype analysis of WT KI and A382T KI iPSCs.

**Additional file 2: Figure S2.** Evaluation of neurite length analysis in motor (A) and sensory (B) neurons derived from control and gene-edited iPSCs. Using the same hydrogen peroxide loading conditions as those used to evaluate CC3, we evaluated the total length of neurites/cell count of motor and sensory neurons as seen by SMI32 or MAP2 immunostaining at DIV45 and 34, respectively. The cells were evaluated separately as cleaved caspase3 negative or positive cells and normalized by the data of cleaved caspase 3 negative control neuron.

**Additional file 3: Figure S3.** Evaluation of TDP-43 localization following oxidative stress. TDP-43 aggregation in the cytoplasm was observed only in A382T-expressing motor neurons, but only to a small extent. Scale bars, 20 μm.

**Additional file 4: Figure S4.** Evaluation of TDP-43 RNA levels in sensory neurons following oxidative stress. There were no significant differences in TDP-43 RNA levels among the three groups.

**Additional file 5: Figure S5.** Evaluation of changes induced by severe oxidative stress. Increasing the concentration hydrogen peroxide and the duration of exposure to hydrogen peroxide resulted in detachment from the dish and cell death in all three groups of motor and sensory neurons, making them unassessable. Scale bars, 200 μm.

**Additional file 6: Figure S6.** (A, B) Evaluation of the alternative splicing function of TDP-43. PDP1 and BCL2L are known TDP-43 alternative splicing targets, and when TDP-43 function is reduced, the proportion of PDP1 splicing variants (PDP1-L/PDP1-S) increases, and the proportion of BCL2L splicing variants (BCL2L-L/BCL2L-S) decreases. In this study, we evaluated the alternative splicing function of TDP-43 by collecting RNA the day after and 5 days after prolonged exposure to low concentrations of hydrogen peroxide. TDP-43 function was not impaired by hydrogen peroxide, and there were no significant differences among the three groups.

**Additional file 7: Figure S7.** (A) TDP-43 RNA sequence in A382T-expressing iPSCs and motor neurons. The presence of mutations was confirmed in the genome but not at the RNA level. (B) Schematic diagram of the gene editing protocol; control, WT (heterozygous), WT (homozygous), and A382T cells. (C) Relative TDP-43 RNA and protein levels in iPSCs. There were no significant differences in TDP-43 RNA and protein levels among the four groups. (C); n = 3 in each of three groups. The data are presented as the mean ± SEM. n.s., not significant. Statistical analyses were performed by one-way ANOVA.

## Data Availability

The datasets used and analyzed during the current study are available from the corresponding authors on reasonable request.

## Abbreviations

ALS: Amyotrophic lateral sclerosis
BCL2L: B-cell CLL/lymphoma 2 like protein
CC3: Cleaved caspase 3
ChAT: Choline acetyltransferase
G3BP1: GTPase-activating protein SH3 domain-binding protein 1
H_2_O_2_: Hydrogen peroxide
iPSCs: Induced pluripotent stem cells
Isl1: Islet 1
Lhx3: LIM homeobox 3
MAP2: Microtubule-associated protein 2
MN: Motor neuron
Ngn2: Neurogenin 2
PDP-1: Pyruvate dehydrogenase phosphatase catalytic subunit 1
SN: Sensory neuron
SOD1: Superoxide dismutase 1
TDP-43: TAR DNA-binding protein 43 kDa
