# Supplementary material for: Specific vulnerability of iPSC-derived motor neurons with TDP-43 gene mutation to oxidative stress
Source: Mol Brain. 2023 Jul 26;16:62. doi: 10.1186/s13041-023-01050-w (PMC10369818; doi:10.1186/s13041-023-01050-w)
Supplement: Supplementary file 1 — Additional file 1: Figure S1. (A) Off-target analysis of WT KI and A382T KI iPSCs. (B) Karyotype analysis of WT KI and A382T KI iPSCs. [file 13041_2023_1050_MOESM1_ESM.pdf]

A

off target	sequence	chromosome position	mismatch	insertion/deletion	score	mutation (WT KI, A382TKI)
1	AAAACTCAATGTTGTATTGGTGG -- hit<br>AAAACTAAAAGCTGTATTGGNGG -- query	Chr2:217389061-217389083	3	No indel	1.53	not detected
2	ACAACTCAAAGCTGGATTGGGGG -- hit<br>AAAACTAAAAGCTGTATTGGNGG -- query	Chr6:83956354-83956376	3	No indel	2.26	not detected
3	AAAACTAAAACCTG-ATTGGTGG -- hit<br>AAAACTAAAAGCTGTATTGGNGG -- query	Chr1:243229130-243229151	1	Del 6	3.11	not detected
4	AAATCTAAAAGCTTTTTTGGAGG -- hit<br>AAAACTAAAAGCTGTATTGGNGG -- query	Chr10:99648829-99648851	3	No indel	3.77	not detected
5	AAAACTAAAAGC-GTTTTGGGGG -- hit<br>AAAACTAAAAGCTGTATTGGNGG -- query	Chr20:4654708-4654729	1	Del 8	3.91	not detected

B

WT KI iPSC line

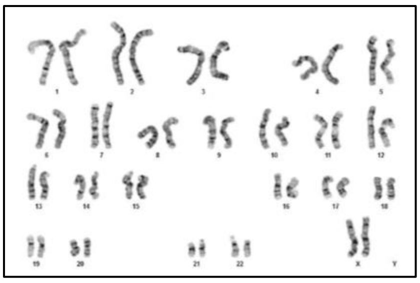

46, XX

A382T KI iPSC line-1

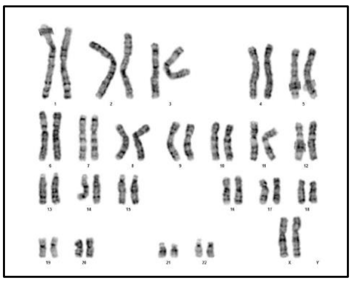

46, XX

A382T KI iPSC line-2

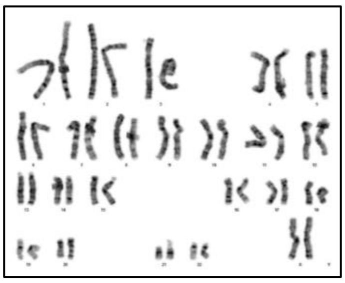

46, XX
