# Supplementary figures and images for: Specific vulnerability of iPSC-derived motor neurons with TDP-43 gene mutation to oxidative stress
Source: Mol Brain. 2023 Jul 26;16:62. doi: 10.1186/s13041-023-01050-w (PMC10369818; doi:10.1186/s13041-023-01050-w)

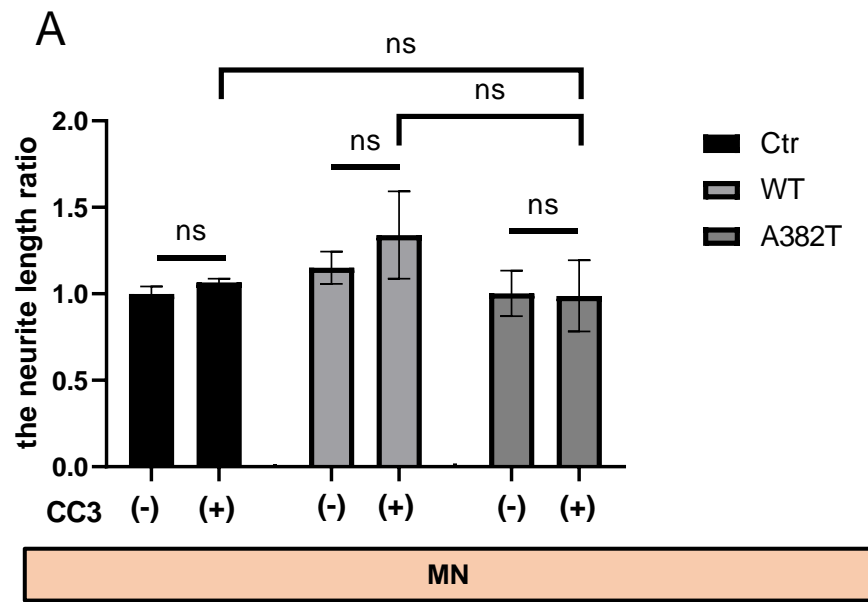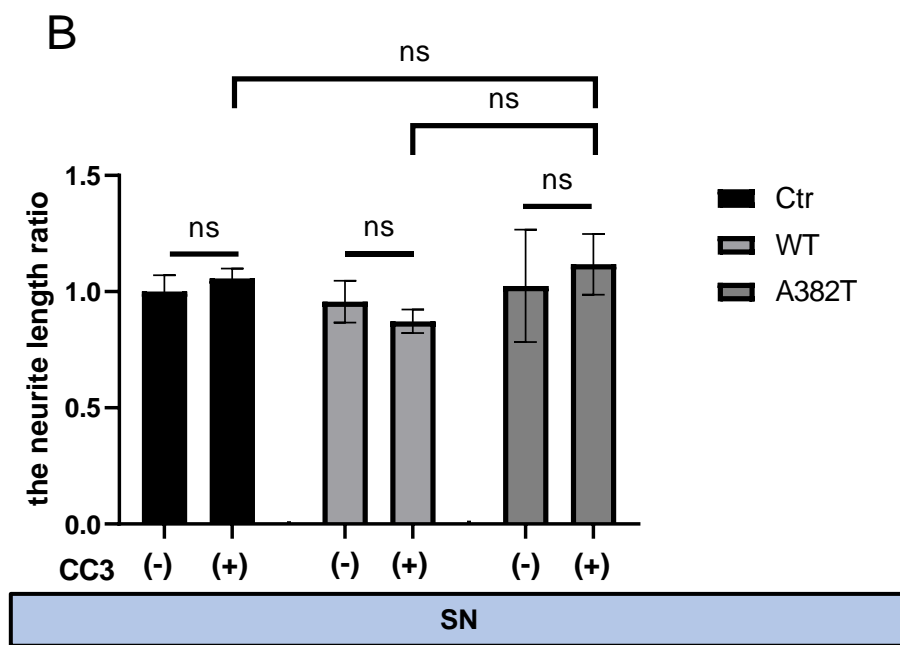

Supplement: Supplementary file 2 — Additional file 2: Figure S2. Evaluation of neurite length analysis in motor (A) and sensory (B) neurons derived from control and gene-edited iPSCs. Using the same hydrogen peroxide loading conditions as those used to evaluate CC3, we evaluated the total length of neurites/cell count of motor and sensory neurons as seen by SMI32 or MAP2 immunostaining at DIV45 and 34, respectively. The cells were evaluated separately as cleaved caspase3 negative or positive cells and normalized by the data of cleaved caspase 3 negative control neuron. [file 13041_2023_1050_MOESM2_ESM.pdf]

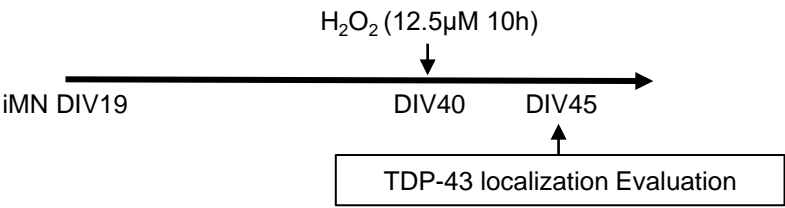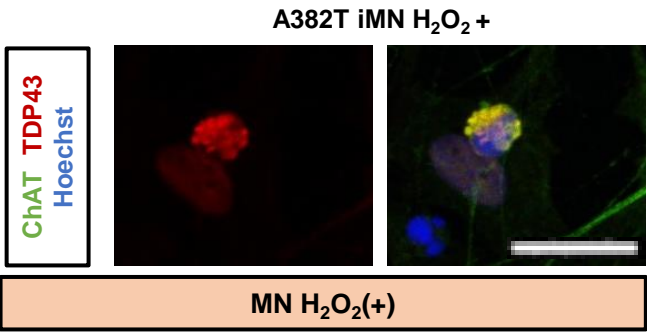

Supplement: Supplementary file 3 — Additional file 3: Figure S3. Evaluation of TDP-43 localization following oxidative stress. TDP-43 aggregation in the cytoplasm was observed only in A382T-expressing motor neurons, but only to a small extent. Scale bars, 20 μm. [file 13041_2023_1050_MOESM3_ESM.pdf]

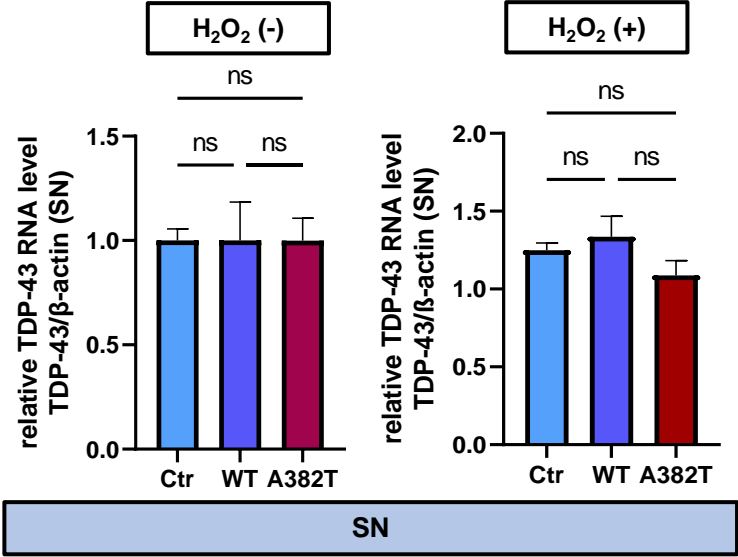

Supplement: Supplementary file 4 — Additional file 4: Figure S4. Evaluation of TDP-43 RNA levels in sensory neurons following oxidative stress. There were no significant differences in TDP-43 RNA levels among the three groups. [file 13041_2023_1050_MOESM4_ESM.pdf]

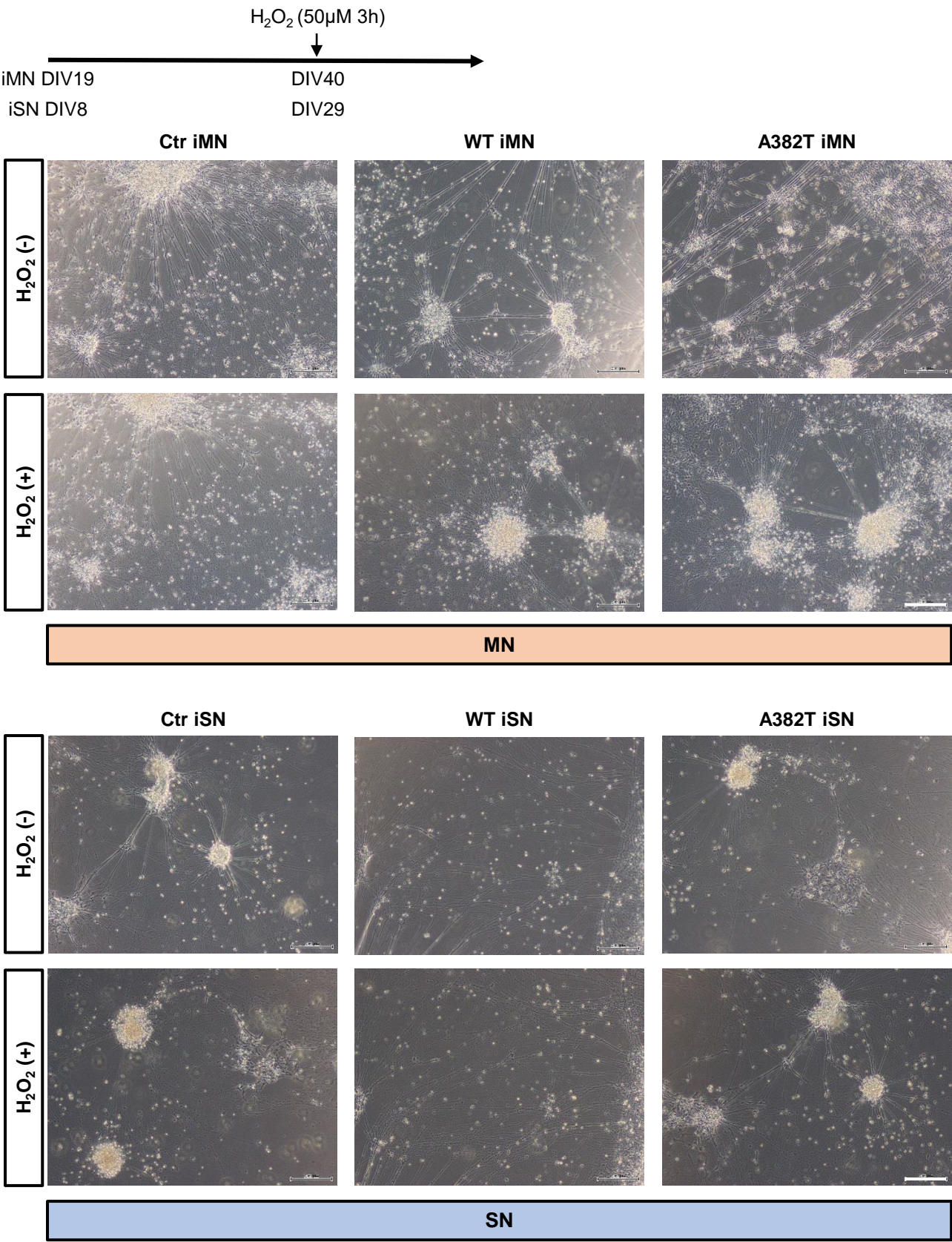

Supplement: Supplementary file 5 — Additional file 5: Figure S5. Evaluation of changes induced by severe oxidative stress. Increasing the concentration hydrogen peroxide and the duration of exposure to hydrogen peroxide resulted in detachment from the dish and cell death in all three groups of motor and sensory neurons, making them unassessable. Scale bars, 200 μm. [file 13041_2023_1050_MOESM5_ESM.pdf]

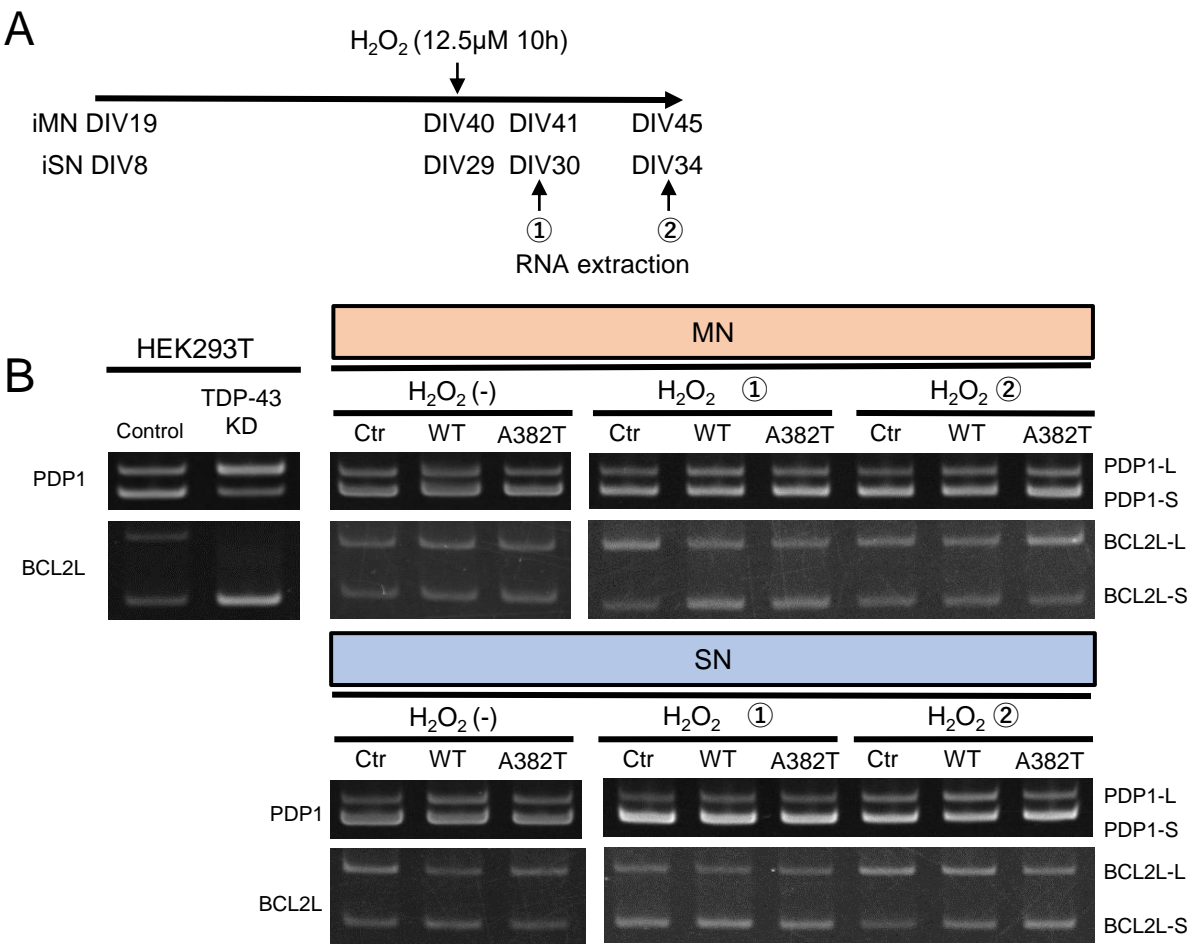

Supplement: Supplementary file 6 — Additional file 6: Figure S6. (A, B) Evaluation of the alternative splicing function of TDP-43. PDP1 and BCL2L are known TDP-43 alternative splicing targets, and when TDP-43 function is reduced, the proportion of PDP1 splicing variants (PDP1-L/PDP1-S) increases, and the proportion of BCL2L splicing variants (BCL2L-L/BCL2L-S) decreases. In this study, we evaluated the alternative splicing function of TDP-43 by collecting RNA the day after and 5 days after prolonged exposure to low concentrations of hydrogen peroxide. TDP-43 function was not impaired by hydrogen peroxide, and there were no significant differences among the three groups. [file 13041_2023_1050_MOESM6_ESM.pdf]

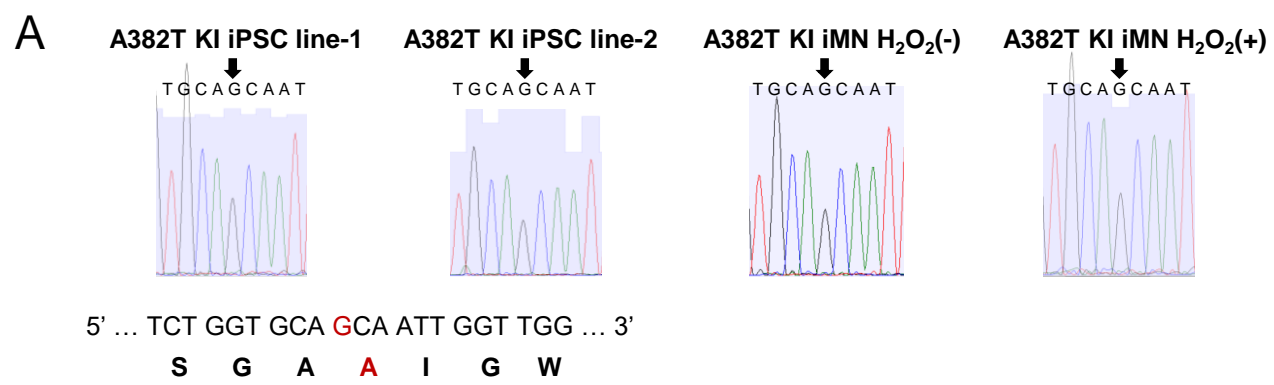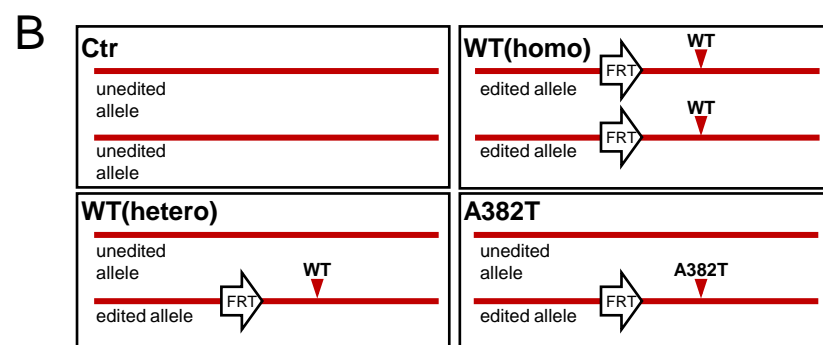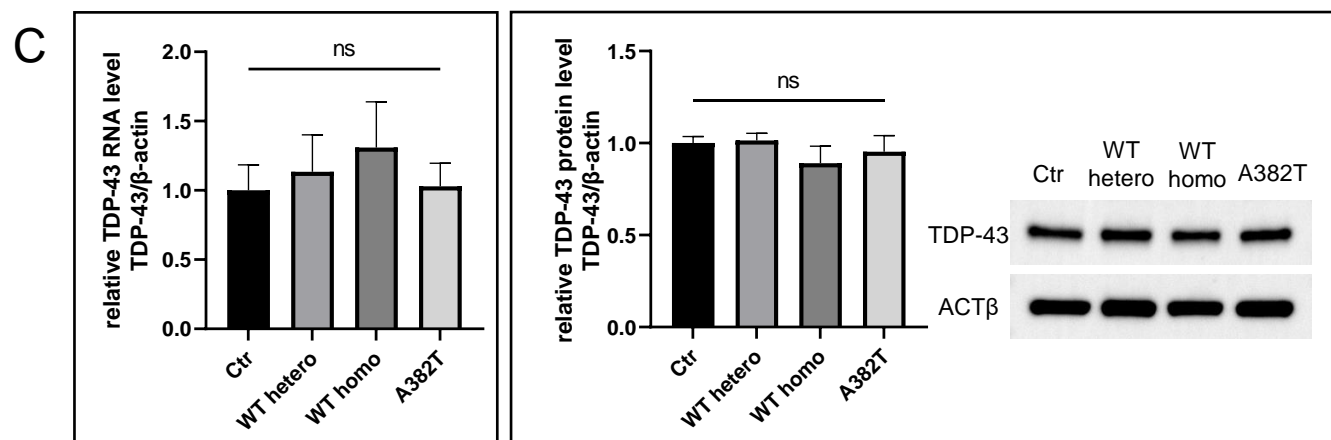

Supplement: Supplementary file 7 — Additional file 7: Figure S7. (A) TDP-43 RNA sequence in A382T-expressing iPSCs and motor neurons. The presence of mutations was confirmed in the genome but not at the RNA level. (B) Schematic diagram of the gene editing protocol; control, WT (heterozygous), WT (homozygous), and A382T cells. (C) Relative TDP-43 RNA and protein levels in iPSCs. There were no significant differences in TDP-43 RNA and protein levels among the four groups. (C); n = 3 in each of three groups. The data are presented as the mean ± SEM. n.s., not significant. Statistical analyses were performed by one-way ANOVA. [file 13041_2023_1050_MOESM7_ESM.pdf]
